# Genotype Profile of *Leishmania major* Strains Isolated from Tunisian Rodent Reservoir Hosts Revealed by Multilocus Microsatellite Typing

**DOI:** 10.1371/journal.pone.0107043

**Published:** 2014-09-09

**Authors:** Wissem Ghawar, Hanène Attia, Jihene Bettaieb, Rihab Yazidi, Dhafer Laouini, Afif Ben Salah

**Affiliations:** 1 Institut Pasteur de Tunis, Service of Medical Epidemiology, Tunis-Belvédère, Tunisia; 2 Institut Pasteur de Tunis, LR11IPT02, Laboratory of Transmission, Control and Immunobiology of Infections (LTCII), Tunis-Belvédère, Tunisia; 3 Université Tunis El Manar, Tunis, Tunisia; University of Brighton, United Kingdom

## Abstract

Zoonotic cutaneous leishmaniasis (ZCL) caused by *Leishmania* (*L*.) *major* parasites represents a major health problem with a large spectrum of clinical manifestations. *Psammomys* (*P.*) *obesus* and *Meriones* (*M.*) *shawi* represent the most important host reservoirs of these parasites in Tunisia. We already reported that infection prevalence is different between these two rodent species. We aimed in this work to evaluate the importance of genetic diversity in *L. major* parasites isolated from different proven and suspected reservoirs for ZCL. Using the multilocus microsatellites typing (MLMT), we analyzed the genetic diversity among strains isolated from (i) *P. obesus* (n = 31), (ii) *M. shawi* (n = 8) and (iii) *Mustela nivalis* (n = 1), captured in Sidi Bouzid, an endemic region for ZCL located in the Center of Tunisia. Studied strains present a new homogeneous genotype profile so far as all tested markers and showed no polymorphism regardless of the parasite host-reservoir origin. This lack of genetic diversity among these *L. major* isolates is the first genetic information on strains isolated from *Leishmania* reservoirs hosts in Tunisia. This result indicates that rodent hosts are unlikely to exert a selective pressure on parasites and stresses on the similarity of geographic and ecological features in this study area. Overall, these results increase our knowledge among rodent reservoir hosts and *L. major* parasites interaction.

## Introduction

Zoonotic cutaneous leishmaniasis (ZCL) represents an important health problem in Tunisia, with a large spectrum of clinical manifestations [1]. *Leishmania* (*L.*) *major*, the causative agent of ZCL is transmitted by the bite of a sand fly vector belonging to the *Phlebotomus* gender and spreading at the Center and the South of the country. Rodent species represent the most important reservoir hosts of this disease [2].

Several studies demonstrated that in some endemic areas, different rodent species are frequently encountered. However, only few species were infected with *Leishmania* parasites. Previous studies using different diagnostic tools showed that *Leishmania* infection prevalence was variable among these rodents [3]–[9]. *Leishmania* parasites might establish in different rodent reservoir hosts with a selective way. In Tunisia, *Psammomys* (*P.*) *obesus* and *Meriones* (*M.*) *shawi* were proven to be the most important rodent reservoir hosts of these parasites [2], [10]–[12], with a wide range of infection prevalence [2], [11], [13]–[16].

In addition to reservoir host selective pressure and factors, parasites might influence the course of the infection. However, *L. major* parasites show a small range of genetic heterogeneity using PCR based methods such as sequencing, restriction fragment length polymorphism analysis and single strand conformation polymorphism analysis [17], [18]. Therefore, markers of higher discriminatory power are needed for population genetic studies and the differentiation of closely related *L. major* parasites.

Microsatellites display a considerable polymorphism for the reason of variation in the number of unit repeats. This high variability makes them the most useful molecular markers available to date for use in genetic typing of individuals [19], population genetic studies and the differentiation of closely related parasites [20]–[22].

The aim of the present study was to analyze the genetic diversity among *L. major* strains isolated from natural reservoir hosts: *Mustela* (*M.*) *nivalis*, *P. obesus* and *M. shawi* in Central Tunisia by applying multi-locus microsatellite typing (MLMT). Gained information might be important to evaluate the interaction between parasites and their animal reservoir hosts during the expansion of ZCL disease and can lead to significant advances in our understanding of processes affecting *L. major* parasites spreading.

## Materials And Methods

### Parasites

Forty strains of *L. major* were isolated between 2009 and 2010 from three reservoir host species: *P. obesus*, *M. shawi* and *M. nivalis*. Animals were captured in Sidi Bouzid, an endemic region for ZCL in Tunisia, as previously described by [2], [23]. These strains derived from the Service of Medical Epidemiology, Pasteur Institute of Tunis.

**Figure 1:**
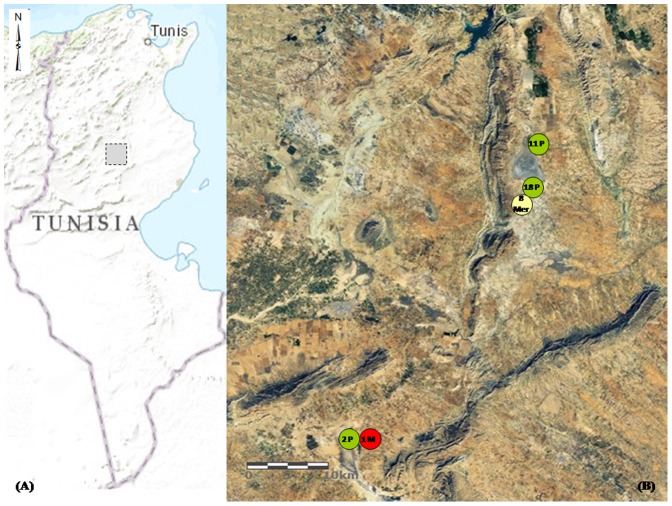
Geographical distribution of strains isolated from the Central Tunisia study area. Panel A shows the square delimited study area in the Governorate of Sidi Bouzid (in gray). Panel B represents the land satellite image of the study area showing the distribution of animal reservoir hosts from which the *L. major* strains were isolated. Isolates from *P. obesus* origin were noted P and colored in green, from *M. shawi* origin were noted Mer and colored in yellow, and from *M. nivalis* was noted M and colored in red. Spatial data related to the reservoir hosts of these strains were collected using the Global Positioning System (GPS). Satellite imagery: ArcGIS software.

The study included thirty one strains isolated from *P. obesus* (Laboratory codes of these strains: *L. major*/Psa 53, *L. major*/Psa 63, *L. major*/Psa 66, *L. major*/Psa 83, *L. major*/Psa 85, *L. major*/Psa 86, *L. major*/Psa 88, *L. major*/Psa 89, *L. major*/Psa 96, *L. major*/Psa 97, *L. major*/Psa 105, *L. major*/Psa 110, *L. major*/Psa 113, *L. major*/Psa 117, *L. major*/Psa 121, *L. major*/Psa 124, *L. major*/Psa 129, *L. major*/Psa 133, *L. major*/Psa 138, *L. major*/Psa 146, *L. major*/Psa 198, *L. major*/Psa 381, *L. major*/Psa 385, *L. major*/Psa 393, *L. major*/Psa 394, *L. major*/Psa 399, *L. major*/Psa 401, *L. major*/Psa 402, *L. major*/Psa 422, *L. major*/Psa 430 and *L. major*/Psa 459), eight strains isolated from *M. shawi* (Laboratory codes of these strains: *L. major*/Mer 15, *L. major*/Mer 16, *L. major*/Mer 109, *L. major*/Mer 114, *L. major*/Mer 116, *L. major*/Mer 128, *L. major*/Mer 138 and *L. major*/Mer 145) and one strain isolated from *M. nivalis*, an accidental reservoir host of *L. major* (WHO code for this strain: *L. major* MMST/TN/2009/NEMS) [2], [23]. Among the 31 strains of *P. obesus*, 11 were isolated from rodents captured at El Mnara foci, 18 at El Khbina foci and two at Ouled Mhammed foci. Strains from *M. shawi* were all isolated from El Khbina foci, while the only strain from *M. nivalis* was isolated from Ouled Mhamed foci. Geographical localization of these foci was described in [2], [23] and is shown in Figure 1.

### DNA extraction

Promastigotes corresponding to each isolate were harvested on the sixth day of culture and a pellet of 2×10^8^ parasites of each isolate was conserved at −20°C until use. DNA extraction was performed using the Qiagen kit according to the manufacturer instructions. DNA of the reference strain (MHOM/IL/1980/Friedlin) which genome is completely sequenced was kindly provided by Prof. Gabriele Schönian, Microbiology and Hygiene Institute (Charité Universitätsmedizin Berlin, Germany).

### ITS1 PCR-RFLP

ITS1 PCR–RFLP was used for the species identification as previously described [24]–[26]. ITS1 sequence was amplified for all strains using LITSR/L5.8S primer pair. Final volume of PCR mix was 50 µl containing 1.5 mM Mg2+, 0.2 mM of each dNTP, 25 pmol of the forward primer (5′ CTGGATCATTTTCCGATG 3′), 25 pmol of the reverse primer (5′ TGATACCACTTATCGCACTT 3′), 1 U of the Taq polymerase and 20 ng of each DNA sample. PCR reaction was performed in a Biometra thermocycler (Biometra, Germany) with an initial denaturation at 95°C for 2 min followed by 33 cycles of (i) denaturation at 95°C for 20 sec, (ii) annealing at 53°C for 30 sec and (iii) extension at 72°C for 1 min. A final extension cycle at 72°C for 6 min was then applied. For each experiment DNA from *L. major*, *L. tropica and L. infantum* species were used as controls and ddH_2_O was used as negative control. The presence of a specific DNA amplified fragment of 300–350 bp has been performed on 2% agarose gel. Subsequently, 17 µl of the PCR product of each amplified DNA, including the controls, were further digested with a total volume of 5 µl of the restriction mixture containing 2.5 µl of ddH_2_O, 1.5 µl of the NE Buffer2 10× concentrated and 1 µl (10 U) of the endonuclease Hae III. After incubation at 37°C, products were visualized under UV in a 2% Metaphor Agarose gel.

### MLMT-PCR

Microsatellite sequences were amplified in a volume of 25 µl containing 1.5 mM Mg2+, 0.2 mM of each dNTP, 5 pmol of each primer, 0.5 U of the *Taq* polymerase and 10 ng of each DNA sample [21]. PCR reaction was performed in a Biometra thermocycler with an initial denaturation at 95°C for 5 min. 35 cycles of denaturation at 95°C for 30 sec, annealing at the specific primer annealing temperature [21] for 30 sec and extension at 72°C for 1 min, are then applied. 35 cycles were finally followed by an extension cycle at 72°C for 6 min. In each experiment, we used DNA of *L. major* MHOM/IL/1980/Friedlin as a reference strain and ddH_2_O as a negative control. Presence, specificity and quality of PCR product were then checked in a 2% agarose gel.

### MLMT-PCR analysis

Exact size of the PCR fragments was established by using a capillary sequencer and the Gene Mapper software v 3.2 (ABI). Fluorescence labeled PCR products were separated using a gel capillary electrophoresis. In some cases, a double peak with only one base difference was obtained due to an A-overhang created during PCR. Hence, only one peak was scored. Fragment sizes for each isolate assessed for the ten microsatellite markers were estimated using the Gene Mapper software and the repeats number was calculated based on the number of repeats of the respective marker in *L. major* MHOM/IL/1980/Friedlin strain (considered as theoretical size). Repeats numbers estimated for the ten loci were assembled into a multilocus microsatellite profile for each used isolate [21], [27], [28].

### Data analysis

Microsatellite based genetic distances were calculated using MSA and POPULATIONS softwares by applying different distance measures appropriate for microsatellites [21]. MSA software package 4.05 (http://i122server.vu-wien.ac.at/MSA/MSA_WIN.html) provides a variety of format outputs used for the analysis of microsatellite data [29]. The distance matrices generated by POPULATION software v1.2.32 (http://www.bioinformatics.org/groups/?group_id=84) allowed the construction of a Neighbor-Joining (NJ) tree with bootstrap values through the Dps (proportion of shared alleles) measure method to analyze the MLMT results using the MEGA 4 tool v 4.0.2 [30], [31].

STRUCTURE software [32] was applied to calculate microsatellite genetic distances according to the proportions of shared allele distance measurements. The program was run as previously described [21]. Individuals can be assigned to multiple clusters with a number of populations (K) ranging from one to 10. Ten runs were performed for each K and the most probable number of populations corresponds to the peak of delta K (ΔK) graph set by plotting ΔK values of K. Descriptive analysis of microsatellite loci including mean number of alleles (MNA), observed (Ho) and expected (He) heterozygosities was performed using GDA [21], [27].

## Results

Amplification of ITS1 sequences of studied isolates, including *L. major*, *L. tropica* and *L. infantum* as reference strains, generated 300–350 bp fragments. After Hae III restriction of ITS1 sequences, all rodent reservoir isolates as well as *L. major* reference strain showed two DNA fragments of 130 and 200 bp corresponding to *L. major* species.

### Microsatellite analysis

MLMT PCR was performed on all strains using a set of ten informative microsatellite markers. For each one of the latter, PCR products of studied strains were controlled on 2% agarose gel and showed the same molecular weight (data not shown).

Theoretical size of amplified fragments obtained show a difference between Friedlin reference strain and isolates obtained from Tunisian rodents in eight, out of ten microsatellites (Table 1). Tunisian strains had the same size as Friedlin for two markers (27GTG and 36GTG), rather. On the other hand, all these isolates showed the same amplified fragment size whether they were obtained from *P. obesus*, *M. shawi* or *M. nivalis* species indicating a homogeneous genotype for all isolated strains.

**Table 1:** Theoretical sizes and repetition numbers among the ten-microsatellite loci in Tunisian studied isolates.

Microsatellites Markers	Theoretical sizes	Repetitions numbers
4GTG	**68**	**6**
27GTG	77	9
36GTG	77	9
39GTG	**59**	**3**
45GTG	**80**	**10**
1GC	**66**	**8**
28AT	**70**	**10**
71AT	**70**	**10**
1GACA	**71** *	**5**
1CA	**115**	**28**

*: New allele not previously described.

Accordingly, MLMT did not reveal any polymorphism within these strains. This indicates that the 40 isolated strains of *L. major* are identical at studied microsatellites level, regardless of their reservoir hosts and their origins.

In order to position Tunisian rodents isolates within the world global context, their MLMT genotype profile was compared to those of 15 *L. major* rodent strains collected from different geographic origins i.e., Turkmenistan, Turkestan, Uzbekistan, Kenya, Iran, Israel and Saudi Arabia [21]. This comparison indicates that Tunisian isolates included in the present study have a unique/new MLMT genetic profile (Table 1).

Indeed, among the 55 rodent strains showing ten genotype profiles, the mean heterozygosity (He) expected under assumption of the Hardy-Weinberg equilibrium was 0.619 with a maximum of 0.8 in 45GTG marker and a minimum of 0.189 in 1GC marker. The mean observed heterozygosity among loci (H_0_ = 0) clearly showed that *L. major* isolates included in this study are exclusively clonal. The mean inbreeding coefficient (Fis = 1) indicated a high inbreeding within loci and the absence of heterozygotes in the investigated strains and all worldwide rodents strains (Table 2).

**Table 2:** Descriptive statistics revealing genetic characteristics and variation of the ten microsatellite loci detected in the population of 55 *L. major* strains isolated from Tunisian rodents and worldwide.

Locus	N	NA	He	Ho	Fis
4GTG	10	2	0.505263	0.0	1.0
27GTG	10	3	0.610526	0.0	1.0
36GTG	9	5	0.758170	0.0	1.0
39GTG	9	4	0.758170	0.0	1.0
45GTG	10	5	0.8	0.0	1.0
1GC	10	2	0.189474	0.0	1.0
28AT	10	4	0.610526	0.0	1.0
71AT	10	4	0.673684	0.0	1.0
1GACA	10	3	0.568421	0.0	1.0
1CA	10	5	0.715789	0.0	1.0
All	9.8	3.7	0.619002	0.0	1.0

N, number of genotypes; NA, number of allele per locus; Ho, observed heterozygosity; He, expected heterozygosity; Fis, inbreeding coefficient.

### Construction of distance trees

**Figure 2:**
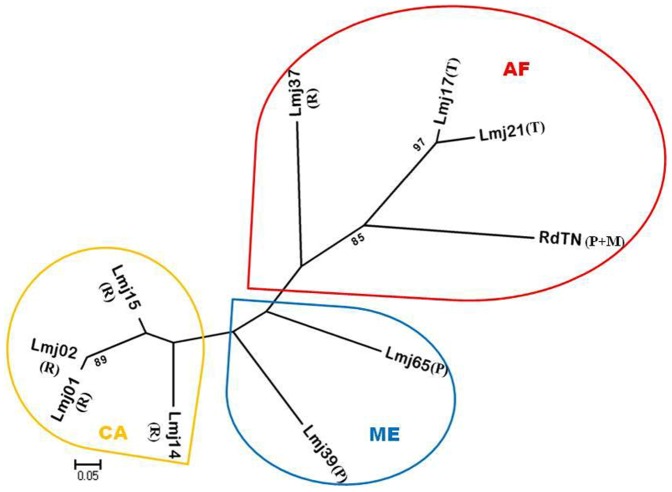
Neighbour-joining tree inferred from the Dps distances calculated for 55 *L. major* strains isolated from different rodents (40 Tunisian and 15 from other geographic origins) according to the 10 microsatellites analyzed. Strains isolated among *P. obesus* (P), *M. shawi* (M), *Tatera sp.* (T) and *R. opimus* (R) were classified into 10 genotypes Lmj01, Lmj02, Lmj14, Lmj15, Lmj17, Lmj21, Lmj37, Lmj39, Lmj65 (as described in[21]) and RdTN from Africa (AF), Middle East (ME) and Central Asia (CA). RdTN indicates the genotype obtained from Tunisian reservoirs. Results are shown as radial tree where the percentages (under 80%) with which a branch is supported in 1000 bootstrap replications are indicated.

The neighbor-joining tree assigned 55 (40 Tunisian and 15 worldwide) *L. major* strains of rodent to three main genetic groups, i.e., one with strains from Central Asia, where Uzbekistan's strain forms a unique cluster, the second group with several clusters of strains from Africa and the third with strains from Middle East (Figure 2). It is noteworthy that this is the first description of *L. major* genotype profile on *M. shawi*.

### Population structure

**Figure 3:**
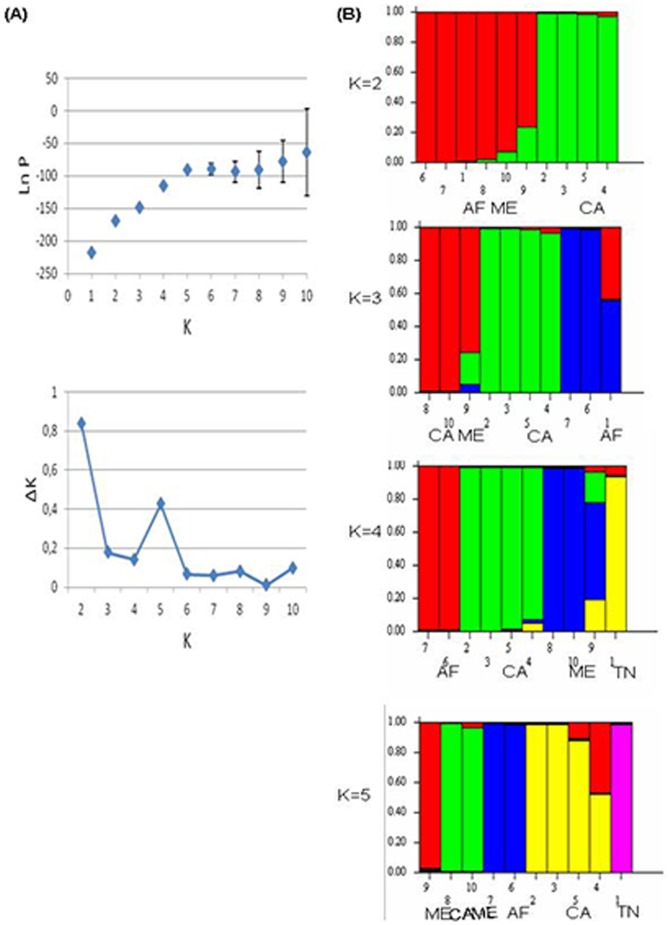
Estimated population structure of 55 *L. major* strains isolated from different rodents strains (40 Tunisian and 15 from other geographic origins) analyzed by STRUCTURE software. A: Likehood number of populations. B: Plots showing the estimated membership coefficient (Q) of each strain. A vertical line represents every strain with one or more colors depending on its degree of membership to one or more clusters. Structure was run 10 times for each assumed number of populations (K from 1 to 10) and plots obtained for K = 2 to K = 5 are represented. Strains were clustered according to their geographic origin i.e., Africa (AF), Central Asia (CA), Middle East (ME) and Tunisia (TN) for the 40 isolates of this present study).

Bayesian model-based clustering approach implemented in STRUCTURE realized to the 55 *L. major* strains (grouped in 10 different genotypes) identified 2 genetic clusters at K = 2. The first one contains all African and Middle East isolates and the second one contains Central Asia isolates. Interestingly, at K = 3, Middle East strains were clustered independently from African ones (including Tunisian strains) and at K = 4, African isolates were classified into two independent clusters i.e., where the Tunisian isolates cluster independently from the other African strains (Figure 3). All these results corroborate those obtained by genetic distance.

## Discussion

To our knowledge, this is the first study in North Africa investigating by MLMT the genetic diversity among *L. major* strains isolated from rodent reservoir hosts, captured in a single endemic area of ZCL in Central Tunisia. We used ten informative microsatellite markers to study the micro-heterogeneity of 40 *L. major* strains of isolated from three different host reservoirs in Central Tunisia (*P. obesus*, *M. shawi* and *M. nivalis*).

No MLMT polymorphism was shown among all tested isolates indicating the absence of genetic variability among *L. major* strains obtained from these different animal reservoir hosts.

MLMT technique is one of the most relevant new molecular and epidemiological tools in regards to its performance for classification and phylogenetic analyzes of *Leishmania* populations at species and even strain levels [33]. This technique revealed interesting genetic variability among all *Leishmania* species as showing for *L. infantum* strains isolated from various areas of Tunisia [34] and *L. donovani* strains [35]. So far there are only two studies that applied MLMT on *L. major* population. These studies showed that this method is promising in the evaluation of refined intra-species genetic polymorphism [21], [22]. In fact, other studies already reported that *L. major* parasites present a lesser amount of polymorphism than other *Leishmania* species [20], [36]–[38]. In addition, it was also shown a correlation between genetic diversity of *L. major* parasites and their geographic origin [21], [22]. This relationship (geographic dependence) was explained by the genetic diversity of parasite vectors [39] and/or rodent reservoir species present in study areas. It should be noted that these assumptions have never been verified previously.

Although carried by three different rodent reservoir species, all our Tunisian *L. major* strains were genetically identical. The restricted geographical area origin might explain their homozygosis and homogeneity. This result corroborates those showing absence of heterogeneity among strains isolated from restricted geographical areas in human and reservoir hosts [21], [22].

This lack of genetic polymorphism in *L. major* isolates might be explained by the development of a specific and long-term adaptation to their host reservoirs in this specific study area. Indeed, it was shown that species isolated from the great gerbils *R. opimus* in Central Asia fail to infect Middle Eastern *P. obesus* rodent host and those isolated from the latter fail to infect Central Asian *R*. *opimus* rodents [17]. This non-permissibility, apparently driven by the co-evolution with the host reservoir, correlates with genetic and biological variations of *L. major* strains isolated from these two regions.

The genetic homogeneity observed among studied strains is however not established yet for parasite isolates obtained from ZCL human patients living within the present study area (personal communication). It is worthy to note however that strains isolated from different involved hosts in the life cycle of *Leishmania* parasites can show a distinct genetic variability [40], [41]. Indeed, these two studies conducted in Morocco and in Southern Spain foci showed that *Leishmania* strains isolated from vectors are distinct from those isolated from their vertebrate host.

ZCL depends on a wide variety of ecological and biological factors and hence geography might affect the distribution of vectors and rodent reservoirs. A recent study has showed that genetic diversity of *L. major* strains belonging to two different endemic areas separated by a chain of mountains [22]. In our case, only a salt lake separates reservoirs location from where *L. major* strains were isolated, and the whole area surrounded by two mountain chains. This might explain the genotypic homogeneity identified for these isolates but this is remains a preliminary finding that requires to know the overall genetic variability of *L. major* in Tunisia (and neighboring endemic regions in other countries) including, besides animal reservoir samples those from sandflies and humans, not only from a particular small region.

Interestingly, when comparing the genotype of Tunisian isolates included in the present study to the genotype of other rodent isolates from the same species described elsewhere [21], the neighbor joining tree allowed their clustering into different populations according to their geographic origin. Indeed, strains from Africa, Central Asia and Middle East were separated in three distinct populations. Although separately clustered, the Tunisian genotype was assigned to the global African rodent *L. major* population, confirming the strong correlation between the microsatellite profile and the geographic origin of each isolate. Besides, our results confirm the homogeneity of the genotype profile observed among strains isolated from rodent reservoirs hosts at the old world range [21], whereas human strains show an important rate of heterogeneity [21], [22]. Furthermore, we illustrate a new profile among isolates from *P. obesus* species and the first one among isolates from *M. shawi* and *M. nivalis* species in addition to the two profiles among *P. obesus* and *Tatera sp.* strains as well as those five found on *R. opimus* strains, previously described [21].

Given the strong homogeneity among the population studied in this work and their difference with isolates from different origins, a larger sample of strains from different ZCL endemic area is needed to better characterize genetic diversity within Tunisian *L. major* strains isolated from rodent reservoir hosts.
